# Aberrant Dynamics of Regional Coherence Measured by Resting-State fMRI in Children With Benign Epilepsy With Centrotemporal Spikes (BECTS)

**DOI:** 10.3389/fneur.2021.712071

**Published:** 2021-12-15

**Authors:** Lin Jiang, Xuejin Ma, Heng Liu, Ji Wang, Jiaren Zhang, Guoming Zhang, Shiguang Li, Tijiang Zhang

**Affiliations:** ^1^Department of Radiology, The First People's Hospital of Zunyi (The Third Affiliated Hospital of Zunyi Medical University), Zunyi, China; ^2^Department of Radiology, Medical Imaging Center, Affiliated Hospital of Zunyi Medical University, Zunyi, China

**Keywords:** benign epilepsy with centrotemporal spikes (BECTS), intrinsic brain activity, resting-state functional magnetic resonance imaging, dynamic regional homogeneity, regional homogeneity

## Abstract

**Objective:** To explore the dynamic features of intrinsic brain activity measured by fMRI in children with benign epilepsy with centrotemporal spikes (BECTS) and examine whether these indexes were associated with behaviors.

**Methods:** We recruited 26 children with BECTS (10.35 ± 2.91 years) and 26 sex-, and age-matched (11.35 ± 2.51 years) healthy controls (HC) and acquired resting-state functional magnetic resonance imaging (rs-fMRI) and behavioral data. Dynamic regional homogeneity (dReHo), including mean and coefficient of variation (CV) metrics derived from the rs-fMRI data, and were compared between the BECTS and the HC groups.

**Results:** Significantly decreased mean dReHo in bilateral supramarginal gyrus, left middle temporal gyrus (MTG.L), left postcentral gyrus and superior occipital gyrus were found in children with BECTS. Meanwhile, increased CV of dReHo in MTG.L and right fusiform in children with BECTS was revealed compared with HC. Further analyses of functional connectivity revealed decreased global signal FC existed in similar regions, linked with linguistic, social cognition, and sensorimotor processes, in children with BECTS compared with HCs. Moreover, the association analyses showed that the CV of dReHo in MTG.L was positively associated with age and a negative correlation was found between mean dReHo of MTG.L and disease duration. Besides, the CV of dReHo in MTG.L was found positively associated with the intelligence quotient (IQ) language scores and full IQ scores in children with BECTS, and the CV of dReHo in the left inferior temporal gyrus and Rolandic operculum were positively correlated with IQ operation scores and full IQ scores.

**Conclusion:** Aberrant dynamic regional coherence in sensorimotor, linguistic, and lateral temporal regions suggests dynamical interplay that underlying cognitive performance in children with BECTS, suggesting an intrinsic dynamic mechanism for BECTS.

## Introduction

Benign epilepsy with centrotemporal spikes (BECTS) is an idiopathic focal epilepsy syndrome with spike-wave discharge around the *Rolandic* fissure, and it is the most common form of epilepsy and generally occurs between the ages of 3 and 13 (1). It is so-called “benign” because most patients have a low seizure frequency and have a spontaneous remission. Emerging evidence, however, suggests that the BECTS has strong associations with cognitive impairment, including language (2), attention (3), and intelligence deficits (4), which may lead to long-term effects into adulthood (1, 5).

To investigate the neural mechanisms, a large body of literature has reported structural and functional alterations in BECTS with magnetic resonance imaging (MRI) (6). For example, a simultaneous functional MRI (fMRI)-EEG study has observed disrupted connectivity involving language and sensorimotor networks during interictal BECTS spikes (7). Studies from resting-state fMRI (rs-fMRI) show that the cortical-subcortical involvements contribute to neuropathology beyond the primary focus (i.e., the *Rolandic* fissure), suggesting distributed changes in children with BECTS. The ReHo, a data-driven method, evaluates the synchronization of local spontaneous activity by measuring functional coherence between a voxel and its neighbor voxels (5, 8). Previous studies focused on the static ReHo (sReHo), and found both reduced and increased sReHo in BECTS children (4, 5, 9). Of particular relevance, in consideration of the subgroup of seizure severity and drug-take, Zeng et al. found that both new-onset and chronic BECTS children showed increased sReHo in the left frontal language region and decreased sReHo in the default mode network (DMN), bilateral occipital lobe and cerebellum compared to healthy controls (HC), and increased sReHo in sensorimotor regions in the new-onset group revealed a normalized or even reversed pattern in the chronic group (5).

The sReHo provides a convenient method and it is sensitive to a series of physiological and pathological processes, but rs-fMRI activity describes spontaneous fluctuations of low-frequency signals over a short period of time (i.e., scan time 8min) and traditional stationary measures only characterize average weaken temporal variations. Thus, a method named dynamic ReHo (dReHo) emerged to capture the temporal variations of regional synchronization over time and has been widely utilized to characterize intrinsic activities of brain (10–12).

Until now, however, little literature has documented the dynamic characteristics of local coherence in children with BECTS, some of which might serve as the neural substrate of BECTS or contribute to related functional deficits. Hence, to demonstrate the problem, we measured dReHo by rs-fMRI to explore whether abnormal local synchronization persisted in children with BECTS compared with healthy controls (HC). Besides, the association between clinical behavior and altered dReHo was evaluated. Pedersen et al. proposed a regulatory network in the brain, which can prevent the patients with focal epilepsy from transitioning from interictal to ictal state, reflecting the plasticity and adaptability of the brain (13). Therefore, it is reasonable to hypothesize that a similar dynamic compensatory mechanism between normal and abnormal states in the brain of individuals with BECTS may be observed.

## Materials and Methods

### Subjects

We recruited 26 children diagnosed with BECTS (spike discharges were left-sided in 14 patients, right-sided in 5 patients and bilateral in 7 patients, and disease duration range from 1 to 96 months) and twenty-six sex- and age-matched HC from the Affiliated Hospital of the Zunyi Medical University, Zunyi, China. These data were from our previous publication (6) and the processes of this study were approved through the Medical Ethics Committee of the Affiliated Hospital of Zunyi Medical University. The time intervals between the last seizure and MRI scanning were 7 days in 5 patients, 3 days in 4 patients, 2 days in 12 patients, and 1 day in 5 patients. The inclusion criteria of BECTS were as follows: (a) BECTS diagnosed by a board-certified pediatric neurologist based on International League Against Epilepsy (ILAE) criteria, excluding children with multiple seizure types; (b) normal physical/ neurological examinations; (c) no other disease except epilepsy; (d) age 6~18 years; (e) right-handedness. With clinical and EEG data, diagnoses followed criteria: (a) ILAE criteria; (b) manifestations of partial or secondarily generalized tonic-clonic seizures diagnosed; (c) EEG showing identifiable sharp waves/spikes at centrotemporal regions especially nocturnal interictal epileptiform; (d) 1-10 seizures within the past year. Exclusion criteria were (a) MRI suggesting a progressive structural central nervous system lesion and (b) falling asleep during the rs-fMRI scan. The mean duration of epilepsy from onset to time of scanning was 20.7 months (SD = 20, min = 4, and max = 84).

### MRI Data Acquisition

All MRI data were collected using a GE 3.0-T (HDxt, GE Healthcare) scanner at the Department of the Radiology of the Affiliated Hospital of Zunyi Medical University. Before the scan, each subject was instructed to stay still with their eyes closed but not fall asleep during the scan. Functional images were acquired by the gradient-recalled echo-planar imaging (EPI) sequence (repetition time = 2,000 ms, echo times = 30 ms, inversion time = 900 ms, flip angle = 90°), 30 sections (field of view = 240 × 240 mm^2^, slice thickness = 4 mm with no gap, and voxel size = 3.75 × 3.75 × 4 mm^3^) and 210 volumes were acquired in 423 s. T1-weighted images were captured by the 3D brain volume imaging (BRAVO) sequence (repetition time = 1,900 ms, echo times = 2.1 ms, inversion time = 900 ms, flip angle = 9°), yielding 160 axial slices (field of view = 240 × 240 mm^2^, slice thickness = 1 mm, and matrix = 256 × 256) with an resolution of 0.94 × 0.94 × 1 mm^3^.

### Neuropsychological Assessment

Children with BECTS accepted standardized neuropsychological tests to collect intelligence quotient (IQ) scores, including IQ-language (93.46 ± 14.73), IQ-performance (85.77 ± 14.71), and IQ-overall (69.42 ± 9.52), using the Wechsler Intelligence Scale for Children (WISC-IV) and its four subscales.

### Data Processing

All rs-fMRI images were preprocessed using the Data Processing & Analysis of Brain Imaging (DPABI ver20200401, http://rfmri.org/dpabi) (14). The procedures included removal of the first 10 volumes, slice timing, and realignment for head motion correction with the Friston 24-parameter model (15). Meanwhile, the frame-wise displacement (FD) (16) of each subject was calculated and introduced as a covariate to control the head motion effect. The remaining 200 frames were coregistered with T1 images. The T1 images were segmented into gray matter, white matter, and cerebrospinal fluid (CSF), and then functional images were normalized to the Montreal Neurologic Institute (MNI) space with the Diffeomorphic Anatomical Registration Through Exponentiated Lie algebra (DARTEL) algorithm (17), and at last, normalized images were resliced at a resolution of 3 × 3 × 3 mm^3^. After that, nuisance signals were regressed, including the head motion parameters, averaged signals from CSF and white matter, and with and without global signal. Finally, functional images were temporal bandpass filtered (0.01–0.08 Hz).

### Dynamic ReHo Analysis

Dynamic analysis was performed in DPABI toolbox based on three sliding window methods (the Hamming window, Hann window, and rectangular window) with various window sizes (window size = 10 TRs, 30 TRs, 50 TRs, and 70 TRs) (10). The argument about the fixed window length is that an ideal window should be both short enough to capture dynamic features of brain fluctuation signal and long enough to accommodate low-frequency signals with a good signal-to-noise ratio. Previous studies of dReHo have applied hamming windows (10). In this work, window lengths of 10 TRs, 30 TRs, 50 TRs, and 70 TRs were applied with a shifting step length of 5 TRs, and the functional images were segmented into different windows for the following ReHo analyses with data points within the window (the Hamming window, Hann window, and rectangular window). We computed the mean and standard deviation of Kendall's coefficient of concordance (KCC) of the time series of each voxel and its nearest 26 neighbor voxels (8, 18).

### Global Signal Functional Connectivity Analysis

In addition, to study the effects on the regional connection in children with BECTS, we try to analyze the alterations of global signal functional connectivity (FC) in these children. To calculate global signal FC, the global signal was first defined as an average time course across all voxels. Then, the Pearson correlation coefficients between the global signal and each voxel's time course of all cortical gray matter were calculated. Finally, these coefficients were converted to Z scores by Fisher Z-transformation to produce a voxel-wise FC map of global signal for each subject.

### Statistical Analysis

Two-sample *t*-tests (*p* < 0.05) were used in between-group comparison for age and education, and Chi-square tests (*p* < 0.05) for sex and handedness. The dReHo metrics and global signal FC were compared between groups using two-sample *t*-tests with education and individual mean FD as covariates of no interest. Multiple comparisons were corrected using family-wise error (FWE) correction with cluster-level *p* < 0.05 (voxel-wise *p* < 0.001).

### Associations Between Brain and Clinical Information

Correlation analyses were further applied to explore the relationship between clinical information (age; disease duration; IQ-language, IQ_LAN; IQ-operation, IQ_OPER; IQ-over all, IQ_OVERALL) and dReHo measures (Mean dReHo/CV of dReHo, Mean/CV; CV of dReHo) in children with BECTS. The significant level was set at *p* < 0.05. Considering the significant educational level between the two groups, we also repeated the correlation analyses using partial correlation controlling the education variables.

## Results

### Clinical Data

The demographic and clinical characteristics of 26 BECTS (15 male/11 female, age: 10.35 ± 2.91 years) and 26 HC (15 male/11 female, age: 11.35 ± 2.51 years) are described in Table 1. There was no significant difference in sex (*p* > 0.99), age (*p* = 0.92) or handedness (*p* > 0.99) between the BECTS and HC groups. Significant decreases (*p* = 0.01) in education were observed in children with BECTS.

**Table 1:** Demographic and clinical characteristics of participants.

**Characteristics**	**BECTS (*n* = 26)**	**HC (*n* = 26)**	***P-*** **value**
	**Mean ± SD (min**~**max)**	**Mean ± SD**	
Sex (male/female)	15/11	15/11	>0.99^‡^
Age, years	10.35 ± 2.91	11.35 ± 2.51	0.92^†^
Handedness (right/left)	40/0	30/0	>0.99^‡^
Education, years	4.31 ± 2.77	5.65 ± 2.53	0.01^†^
Duration, months	38.81 (1~96)	-	-
IQ-language scores	93.46 ± 14.73	-	-
IQ-performance scores	85.77 ± 24.71	-	-
IQ-over all scores	69.42 ± 9.52	-	-

*BECTS, benign epilepsy with centrotemporal spikes; HC, healthy control; n, number of subjects; SD, standard deviation; IQ, intelligence quotient*.

‡ *Chi-squared test was used;*

† *Two-sample t test was used*.

### Between-Group Comparison on Dynamical ReHo Without Global Regression

**Figure 1:**
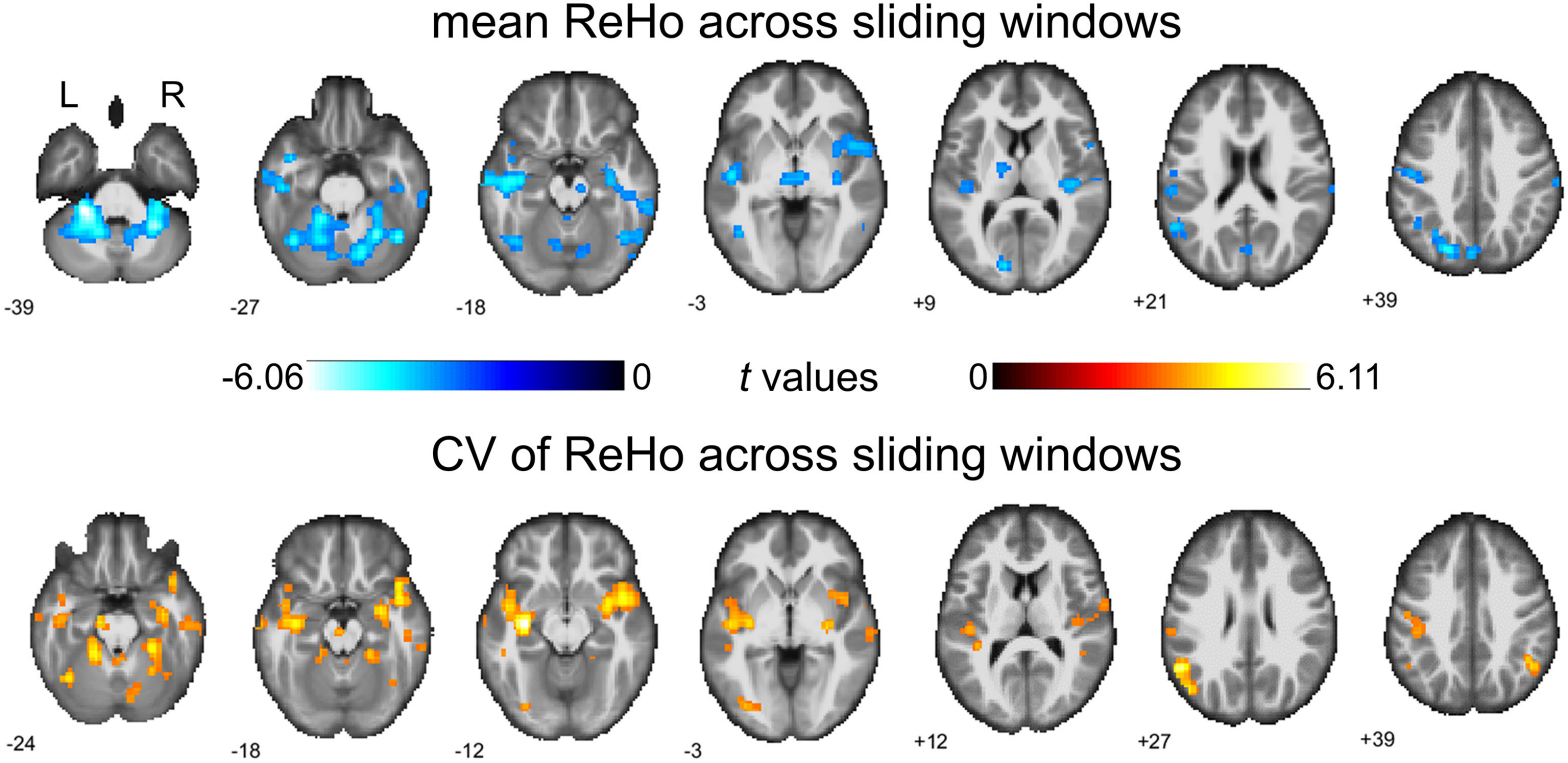
Between-group comparisons of mean dReHo and coefficient of variation (CV) of ReHo across sliding windows (without global signal regression). Both significantly decreased mean dReHo (top row) and increased CV of ReHo (bottom row) were found in the left temporal pole (TPO.L), left middle temporal gyrus (MTG.L). The warm color represents an increase and the cold color represents a decrease when comparing children with BECTS with HCs. These results were voxel-wise *p* < 0.001, cluster level *p* < 0.05, family-wise error (FWE) cluster-level corrected, and minimum cluster > 20 voxels.

We first report the between-group comparison on dReHo measures without global signal regression (GSR). Significant decreases of mean dReHo in the left postcentral gyrus (POG.L), left temporal lobe (including left Heschel gyrus, HES.L; left middle temporal gyrus, MTG.L; left temporal pole, TPO.L), left superior occipital gyrus (SOG.L), left precuneus (PCUN.L), left calcarine gyrus (CAG.L), bilateral supramarginal gyri (SMG.R and SMG.L) and part of the bilateral inferior cerebellum (including left lobules IX, X and right lobule VI) were identified in children with BECTS (Figure 1 and Table 2). Instead, significant increases of the CV of dReHo was demonstrated in the left inferior parietal lobule (IPL.L), left inferior occipital gyrus (IOG.L), bilateral temporal lobes (including right Heschel gyrus, HES.R; left superior temporal gyrus, STG.L, bilateral MTG, bilateral TPO and bilateral inferior temporal gyri, ITG), left hippocampus (HIP.L), right fusiform gyrus (FFG.R), right angular gyrus (ANG.R), right superior cerebellum (including lobules IV-V, VI) and part of the cerebellar vermis (Figure 1 and Table 3).

**Table 2:** Between-group comparison of mean dReHo across sliding windows.

**Brain regions**	**Hemisphere**	**Peak MNI coordinates**			**Voxels**	**Peak *t*-value**
		**X**	**Y**	**Z**		
**Without global regression**						
Cerebelum (X)	L	−27	−39	−39	25,56	−6.059
Cerebelum (VIII)	R	30	−51	−39	2,556	−5.716
Cerebelum (IX)	L	−15	−57	−36	2,556	−5.234
Middle temporal gyrus	L	−48	−15	−18	628	−4.846
Middle temporal gyrus	L	−42	3	−27	628	−4.432
Temporal pole	L	−51	3	3	628	−3.494
Superior occipital gyrus	L	−21	−78	36	264	−4.808
Precuneus	L	−9	−66	51	264	−4.185
Calcarine gyrus	L	0	−72	27	264	−4.068
Calcarine gyrus	L	−12	−87	9	41	−4.296
Middle temporal gyrus	L	−54	−57	24	103	−4.290
Supra marginal gyrus	R	66	−24	33	38	−3.934
Postcentral gyrus	L	−51	−15	39	56	−3.852
Postcentral gyrus	L	−60	−12	21	56	−3.367
Heschls gyrus	L	−42	−24	12	83	−3.798
Supra marginal gyrus	L	−66	−27	30	83	−3.566
**With global regression**						
Cerebellum (VI)	L	−27	−39	−39	72	−4.642
Supramarginal gyrus	R	66	−24	33	62	−4.283
Middle temporal gyrus	L	−66	−15	−15	228	−4.282
Hippocampus	L	−36	−15	−18	228	−3.876
Middle temporal gyrus	L	−51	−30	−12	228	−3.670
Postcentral gyrus	L	−54	−12	36	234	−4.278
Supramarginal gyrus	L	−54	−27	15	234	−3.738
Superior occipital gyrus	L	−21	−78	36	71	−4.256
Cerebellum (Crus 1)	R	45	−60	−24	38	−4.224
Inferior frontal operculum	R	57	9	3	268	−4.211
Insula gyrus	R	42	0	−15	268	−3.981
Fusiform gyrus	R	45	−24	−24	268	−3.780
Cuneus	L	0	−75	27	37	−4.166
Cerebellum (VI)	L	−39	−63	−24	25	−4.030
Superior temporal pole	L	−48	15	−15	36	−3.998
Precuneus	L	−12	−63	54	64	−3.914
Precuneus	R	12	−54	51	28	−3.828
Inferior temporal gyrus	R	60	−36	−21	40	−3.901
Rolandic operculum	R	63	−18	12	64	−3.706
Angular gyrus	L	−48	−69	24	38	−3.641

**Table 3:** Between-group comparison of the CV of ReHo across sliding windows.

**Brain regions**	**Hemisphere**	**Peak MNI coordinates**			**Voxels**	**Peak *t*-value**
		**X**	**Y**	**Z**		
**Without global regression**						
Hippocampus	L	−33	−15	−9	537	6.112
Temporal pole	L	−48	0	−12	537	4.395
Inferior temporal gyrus	L	−48	−21	−30	537	4.163
Middle temporal gyrus	L	−54	−57	27	162	5.412
Cerebellar vermis (X)	–	3	−54	−30	531	4.174
Cerebelum (IV-V)	R	27	−39	−27	212	4.877
Cerebelum (VI)	R	39	−60	−18	212	3.509
Temporal pole	R	45	6	−12	539	4.810
Fusiform gyrus	R	36	−12	−24	539	4.413
Cerebelum (Crus 1)	L	−39	−63	−27	57	4.508
Inferior occipital gyrus	L	−27	−84	3	73	4.379
Angular gyrus	R	48	−57	39	50	4.226
Inferior parietal lobule	L	−42	−30	42	47	4.159
Inferior temporal gyrus	R	57	−21	−27	88	3.838
Superior temporal gyrus	L	−48	−27	15	67	3.827
Middle temporal gyrus	L	−51	−39	−9	23	3.720
Middle temporal gyrus	R	66	−21	−3	20	3.599
Heschls gyrus	R	63	−6	12	22	3.597
**With global regression**						
Insula gyrus	R	45	3	−12	221	5.327
Fusiform gyrus	R	39	−24	21	221	3.446
Cerebellum (IV-V)	L	−21	−45	−27	43	4.941
Postcentral gyrus	L	−45	−18	36	279	4.712
Middle temporal gyrus	L	−54	−18	−3	279	4.323
Middle temporal gyrus	L	−45	3	−27	49	4.628
Middle temporal gyrus	R	66	−36	6	86	4.569
Temporal pole	L	−51	15	−15	34	4.196
Rolandic operculum	R	63	−6	9	70	3.809
Postcentral gyrus	R	63	−21	36	25	3.775
Cerebellum (Crus 1)	R	42	−69	−24	26	3.684

**Figure 2:**
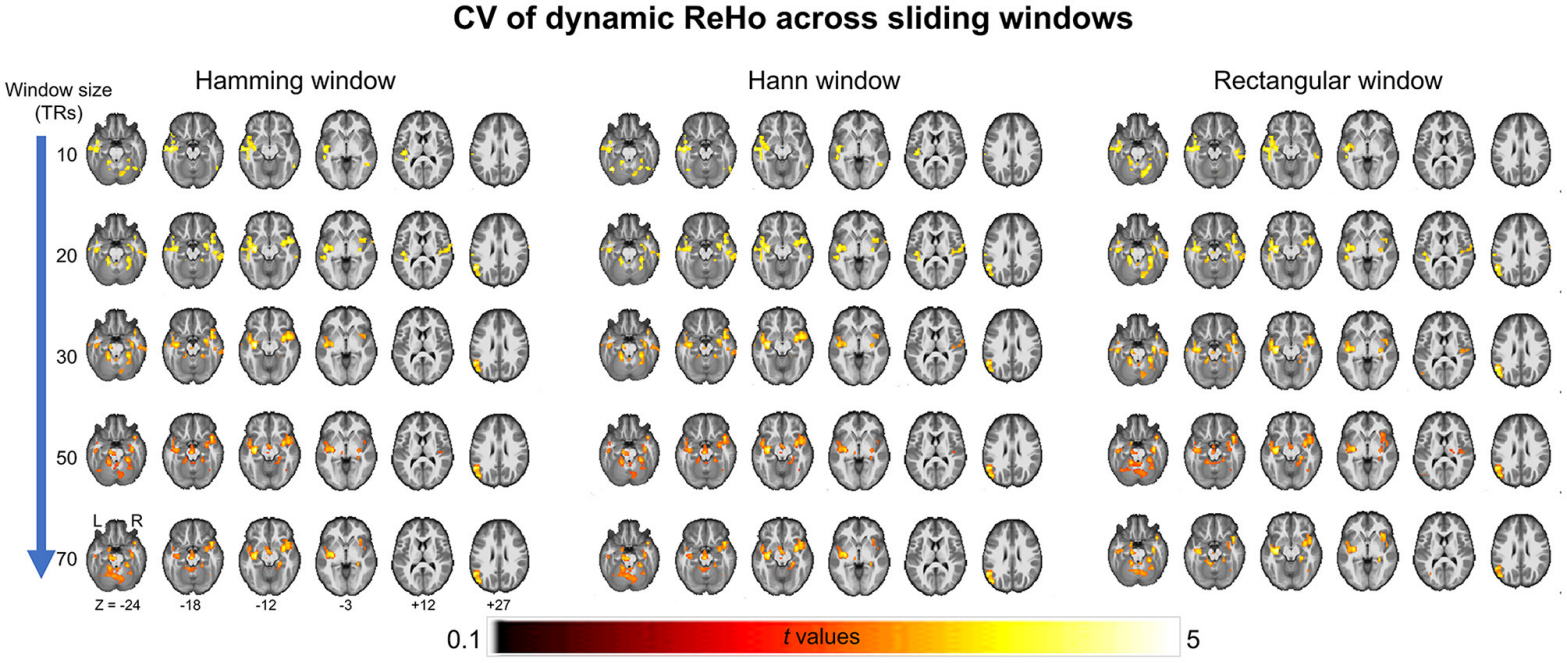
Between-group differences of coefficient of variation (CV) of ReHo across different sliding window methods (the Hamming window, Hann window, and rectangular window) with various window sizes (window size = 10 TRs, 30 TRs, 50 TRs, and 70 TRs).

As a validation, we then report consistent between-group differences across different sliding window methods (the Hamming window, Hann window, and rectangular window) with various window sizes (window size = 10 TRs, 30 TRs, 50 TRs, and 70 TRs) (Figure 2).

### Between-Group Comparison on Dynamical ReHo With Global Regression

**Figure 3:**
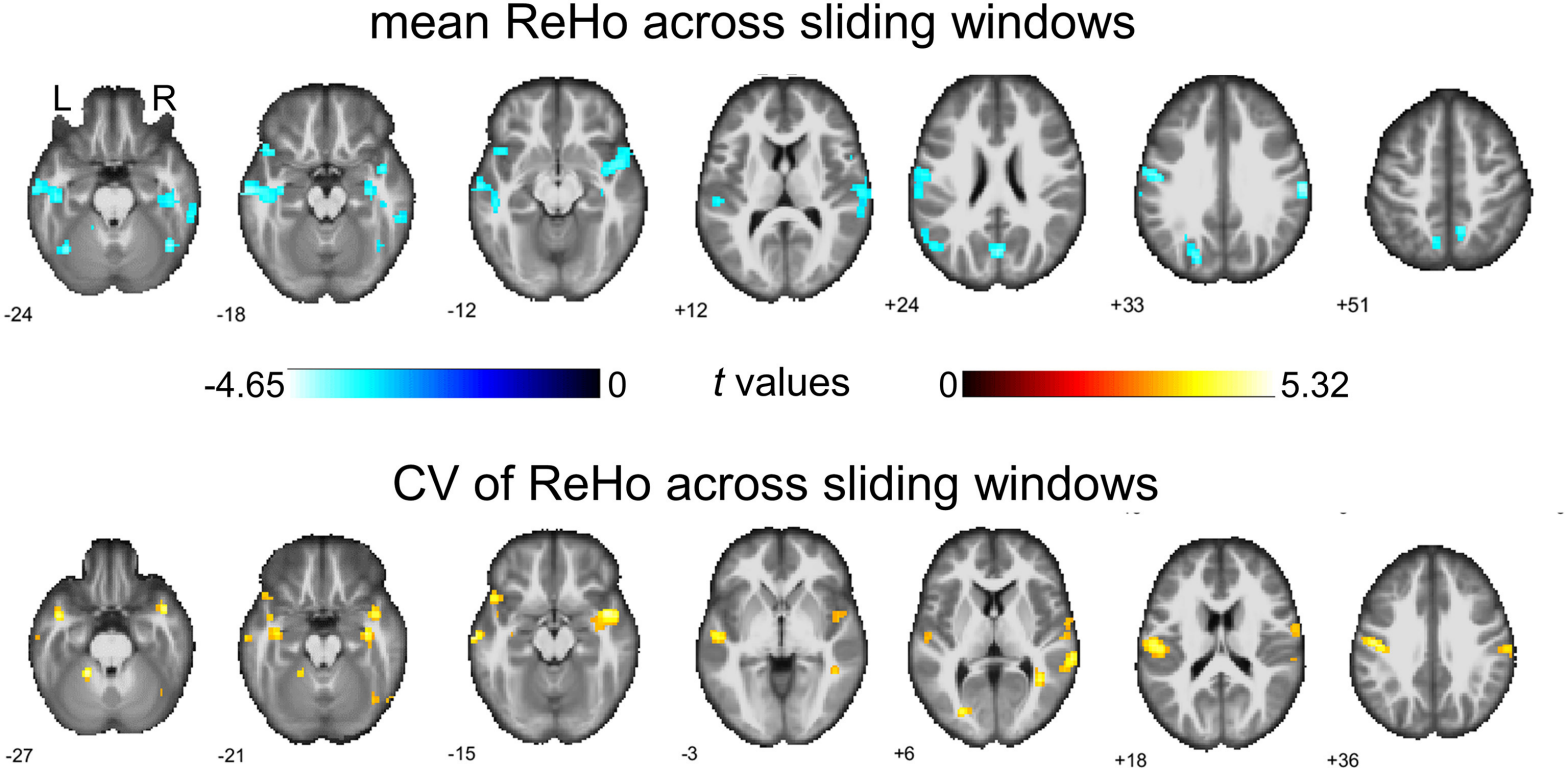
Between-group comparisons of mean ReHo and coefficient of variation (CV) of ReHo across sliding windows (with global signal regression). Both significant decreased mean ReHo (top row) and increased CV of ReHo (bottom row) were found in the right insula gyrus (INS.R), right fusiform gyrus (FFG.R), left postcentral gyrus (POG.L), left middle temporal gyrus (MTG.L), right rolandic operculum/superior temporal gyrus and part of the right cerebellum (lobule Crus1). The warm color represents increases and the cold color represents decreases when comparing children with BECTS with HCs. These results were voxel-wise *p* < 0.001, cluster level *p* < 0.05, family-wise error (FWE) cluster-level corrected, and minimum cluster > 20 voxels.

We then report the between-group comparison on dReHo measures with the GSR. Regions including POG.L, bilateral SMG, bilateral temporal lobe (including MTG.L, TPO.L, and ITG.R), right insular gyrus (INS.R), right inferior frontal gyrus (IFG.R), HIP.L, FFG.R, SOG.L, left cuneus gyrus (CUN.L), bilateral precuneus gyrus (PCUN.R and PCUN.L), ANG.L, right *Rolandic* operculum (ROL.R)/STG.R, and part of the bilateral inferior cerebellum (including left lobules VI and right lobule CRUS1) showed decreases in mean dReHo in children with BECTS compared with HC (Figure 3 and Table 2).

Besides, significant increases were demonstrated in the CV of dReHo in INS.R, FFG.R, bilateral postcentral gyrus (POG.L and POG.R), bilateral MTG, TPO.L, right *Rolandic* operculum (ROL.R)/STG.R, and part of the bilateral cerebellum (including left lobules IV-V and right lobule CRUS1) (Figure 3 and Table 3).

### Between-Group Comparison on Global Signal FC

**Figure 4:**
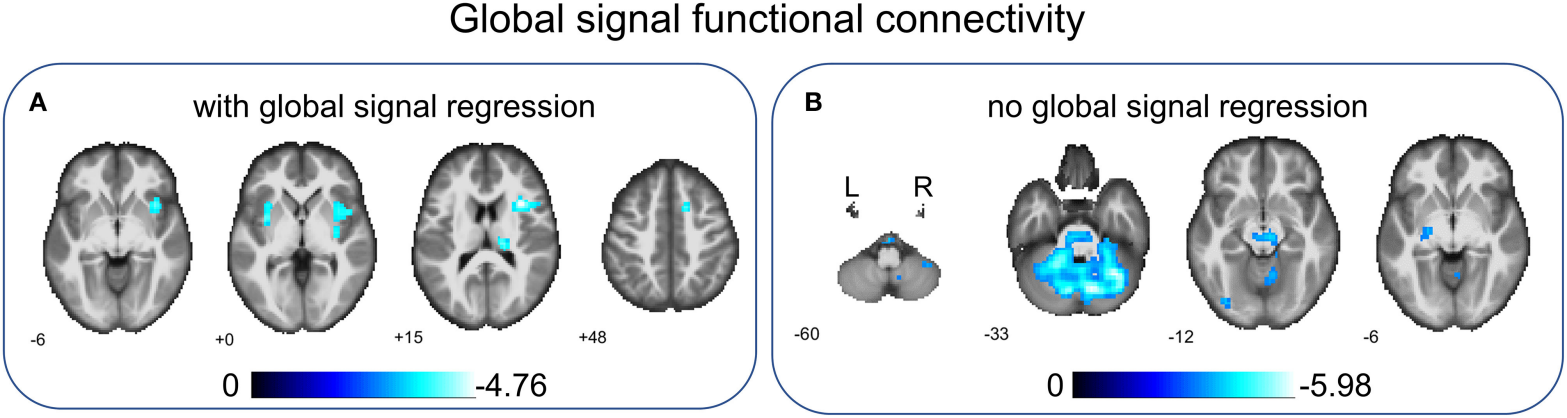
Between-group comparisons of global signal FC with/without global signal regression. After global signal regression, significantly decreased global signal FC was found in the right insular gyrus (INS.R), right thalamus (Thalamus_R), right superior frontal gyrus (SFG.R), and right putamen (Putamen_R) in children with BECTS compared with HCs **(A)**. Before global signal regression, significantly decreased global signal FC was revealed in the inferior occipital gyrus (IOG.L) and part of the bilateral cerebellum (left and right lobule Crus1) in children with BECTS when comparing to HCs **(B)**. These results were voxel-wise *p* < 0.001, cluster level *p* < 0.05, family-wise error (FWE) cluster-level corrected.

Based on the analyses of regional connectivity disturbance in children with BECTS, global signal FC between groups was compared to further explore the neural activity changes over the entire cortex in BECTS. After GSR, children with BECTS showed significantly decreased global signal FC in INS.R, right thalamus (Thalamus_R), right superior frontal gyrus (SFG.R), and right putamen (Putamen_R) (Figure 4A and Table 4) compared with HCs. Without GSR, significant decreases of global signal FC were revealed in IOG.L and part of the bilateral cerebellum (right lobules CRUS1 and left lobule CRUS1) (Figure 4B and Table 4) in children with BECTS compared with HCs.

**Table 4:** Between-group comparison on global signal FC.

**Brain regions**	**Hemisphere**	**Peak MNI coordinates**			**Voxels**	**Peak *t*-value**
		**X**	**Y**	**Z**		
**Without global regression**						
Cerebellum (Crus 1)	R	12	−72	−30	3,090	−5.976
Cerebellum (Crus 1)	R	36	−60	−33	3,090	−5.939
Cerebellum (Crus 1)	L	−9	−75	−30	3,090	−5.751
Inferior occipital gyrus	L	−36	−84	−12	20	−3.969
**With global regression**						
Insula gyrus	R	36	3	0	260	−4.066
Thalamus	R	15	−24	15	30	−4.069
Superior frontal gyrus	R	15	12	48	19	−3.912
Putamen	R	33	−12	0	19	−3.718

### Correlations Between Clinical Data and Behaviors

**Figure 5:**
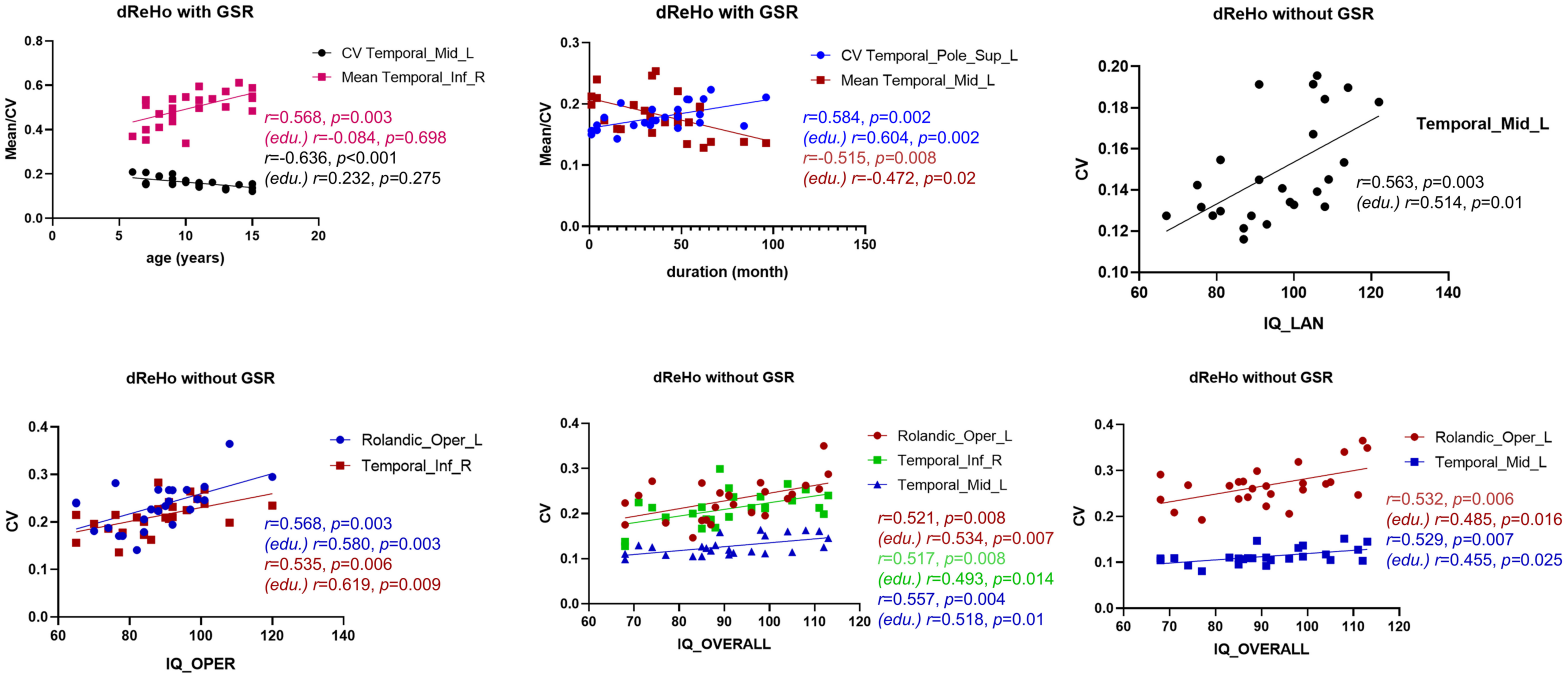
Association analyses. With global signal regression (GSR), the CV of dReHo in the left middle temporal gyrus (MTG.R) was positively associated with age, and a negative correlation was revealed between the mean dReHo of the MTG.R and duration. Besides, a significantly negative association was found between the mean dReHo of the right inferior temporal gyrus (ITG.R) and age, and the CV of dReHo in the left temporal pole (TPO.L) was positively related to disease duration. Without GSR, the CV of dReHo in MTG.L was positively correlated to IQ_LAN and IQ_OVERALL scores in children with BECTS. In addition, the CV of dReHo in the left Rolandic operculum (ROL.L) and ITG.R were both positively associated with IQ_OPER and IQ_OVERALL scores. Results with age regression are also marked (edu.).

To further understand the relationship between dReHo measures and clinical information in children with BECTS, correlation analyses were applied (Figure 5). After GSR, mean dReHo of the ITG.R was positively correlated with age, and CV of dReHo in TMG.L was negatively associated with age. Besides, a significantly negative association was found between the mean dReHo of the MTG.L and disease duration, and the CV of dReHo in TPO.L was positively related to duration. Before GSR, the CV of dReHo in MTG.L was positively correlated to IQ_LAN and IQ_OVERALL scores. Moreover, the CV of dReHo in ROL.L and ITG.R were positively associated with IQ_OPER and IQ_OVERALL scores.

Given the educational delays in children with BECTS, education variables may affect the results of the correlation analyses. Therefore, we repeated the analyses controlling the education variables and found that only significant age effects became no longer significant (Figure 5). Other correlations, although weakened to some extent, were still significant (Figure 5).

## Discussion

We investigated voxel-wise dynamical regional coherence measured with resting-state fMRI to search for abnormal local dynamics that underlie cognitive performance in children with BECTS. Both a decreased mean dReHo and an increased CV of dReHo were revealed in sensorimotor-, language-, and visual-related cortices in children with BECTS with/without global regression, and a reduced global signal FC was uncovered. In addition, a significant correlation between the changed dReHo metrics and clinical data may further confirm the ability of dReHo to serve as a neural marker in the diagnosis of BECTS.

### Dysfunction in BECTS

Emerging studies have documented distant spreading effects of the abnormal discharges in BECTS, which resulted in various functional consequences. Consistent with the previous literature (4, 5, 9), we found significantly decreased mean dReHo in the bilateral SMG, MTG.L, POG.L, and SOG.L (overlap of regions with/without global regression) in children with BECTS compared with HCs.

Short-range connectivity quantified by (d)ReHo is considered as the basis of long-range functional integration across large-scale functional networks and advanced cognition (19). The SOG serves as a visual area in the dorsal attention network, receiving an external stimulus and top-down control to reorient attention to most-concerned information (20). Thus, the decreased mean dReHo in SOG.L represents decreased average intrinsic activity and may contribute to impairments of the goal-directed attention in children with BECTS. Besides, SMG.L is adjacent to Wernicke's area and confirmed to be closely associated with single-word comprehension and semantic memory (21). Decreased local synchronization within SMG.L may suggest poor linguistic skills and subsequent verbal learning dysfunction. The MTG.L is functionally involved in visual, auditory, and language processing (22), and its neuroplasticity may be easily affected by frequently abnormal discharges that could link to social cognition and communication dysfunction. Moreover, according to previous studies (5, 23), POG.L was presumed to be the location of the generator of discharge (24, 25), so our results showing decreased mean dReHo in this area may be attributed to disrupted coherence in children with BECTS. Furthermore, reduced global signal FC was detected in language and working memory related regions (IOG.L and bilateral lobe crus1 of the cerebellum, without global regression) and the transmission pathway of sensorimotor information (INS.R, Thalamus.R, SFG.R, and putamen.R, without global regression) may be correlated with long-term functional disruption in BECTS.

There are two interesting findings worthy of attention. First, different from both increases and decreases of static ReHo described in previous studies (4, 5, 9), only a decreased mean dReHo was revealed in the present study. Although increased connectivity related to epilepsy zones coupling with decreased connectivity in distal networks is widely revealed in focal epilepsy, connectivity patterns might change with disease course (26). By subdividing participants into new-onset and chronic BECTS, Zeng et al. (5) found brain regions showing increased ReHo in the new-onset group may display normalized or even decreased results in the chronic group. The duration of disease in the present study is similar to that in the chronic group, hence, it is reasonable to reveal mean dReHo diminished with duration in part of the cortex (Figure 5), suggesting long-term disrupted affection of disease or positive decreases caused by drugs. Additionally, the aberrant differences mainly appeared in the left side of the brain, which may be caused by the fact that most participants have left epilepsy zones.

In summary, besides the period of ictal/interictal with abnormal discharges, functional abnormality exists in the whole interictal period, and the decreased mean dReHo observed in the aforementioned regions suggests an aberrant connection and integration in language, attention, and sensorimotor circuits, further leading to cognition and behavior dysfunction in children with BECTS.

### Compensated Regulation in BECTS

BECTS is a kind of focal epilepsy, and a large body of studies support it as a ‘global problem’ with the ability to damage functions in both epilepsy focus lesions and distant areas due to the discharge propagation across the whole brain (3, 4, 6, 27–29). Meanwhile, abnormal discharge during both ictal and interictal states can cause long-term brain dysfunction (4, 7). The dReHo provides us with a dynamic view to characterize the local connection within anatomically spatial neighbors, which may reflect spontaneous activities of neuro cells activity with a combination of structure and function (30). Our results show an increased CV of dReHo in MTG.L and FFG.L (overlap of regions with/without global regression) that may display the regulation in children with BECTS. Diminished local connection quantified by decreased mean dReHo uncovers functional abnormalities in sensorimotor, language, and cognition area of BECTS, but the increased CV of dReHo might represent the brain trying to regulate the loss of connection and limit the spread of ictal state. Moreover, increases involving both the left and right cerebral may further reveal an adaptive mechanism to manage global compensation to the disruption in the left connection. And the positive/negative correlation between the CV of dReHo in the MTG.L and duration/age may further reveal an enhanced/weakened compensation effect of the brain (Figure 5), supporting the benign changes to some extent. However, this supposition needs to be further confirmed. On the other hand, it could be speculated that abnormal local synchronization in these areas results in unstable neurofluctuation and leads to a long-term impairment of the related social cognition and sensorimotor networks (1, 2, 22, 23).

### Effect of GSR on Functional Connections in BECTS

A large body of research has shown that global signal may represent an integration of neuronal activities and some noise (31–33). Whether regressing global signal in preprocessing remains elusive and both were utilized in previous studies of ReHo in BECTS (4, 5, 9, 34). Therefore, we calculated results in both situations, and it is interesting that decreased mean dReHo, increased CV of dReHo, and decreased global signal FC exist in children with BECTS compared with HCs in both cases. Even though clusters showing significant differences were different, their locations are linked to similar linguistic, social cognition, and sensorimotor processes. Murphy et al. (35) suggested that the global regression may introduce negative correlations which were presumed to be the biological underpinnings of the organizational feature of the human brain (36, 37). In our study, the number and size of clusters showing significant changes with global regression were less than those without regression. For global signal FC, GSR may censor time points with large global signal magnitude and result in unequally reduced variance between groups, leading to altered between-group comparisons (32, 38). As for ReHo, a previous study has confirmed that the effects of GSR on sReHo were complex and heterogeneous (39). Our results revealed that the sum of all cluster areas showing differences in dReHo measures was reduced after GSR, and the changes in each cluster were different, which proved its heterogeneity. Therefore, other unknown mechanisms and the robustness of GSR on dReHo need to be further explored, and the results with and without global regression should be taken into consideration.

There are several limitations in the current study. First, due to the lack of cognitive tests in the HC, we cannot infer the extent to which the BECTS children may lag behind the HC in IQ scores. Nevertheless, previous literature has reported that BECTS children had lower IQ rankings, although this may still be within the normal range (40). Second, the education of BECTSs is significantly lower than that of the HC group. One source of this difference, we speculated, may be the delay of the normal educational process caused by the disease; on the other hand, it may also reflect cognitive dysfunction and delay in brain maturation in BECTS children, thus it is difficult for them to reach the level of education required for normal age. This speculation, indeed, is consistent with a previous report (40) which suggested that BECTS was accompanied by specific cognitive disorders and low academic achievement. Our association analyses also suggested that education level influenced the age-related changes in dReHo in lateral temporal regions. Third, epileptogenic foci are necessary for the propagation of epilepsy and it varies in our patients, however, we did not further explore the effects of foci location due to the limited sample size. Finally, although most BECTS children involved in this study were treated with medication depending on the severity of the disease, we did not collect sufficient data to investigate the medication-related effects on dynamic features of BECTS neural activities, which should be explored in further study.

## Conclusions

We first delineated the dynamic local coherence in BECTS by dReHo. The results suggested dysfunction in sensorimotor, linguistic, and cognition networks, and we also highlighted the possibility of increased dReHo variability in these regions representing a compensation mechanism for functional problems or the persistent instability of related systems in children with BECTS. Our findings may provide a neuropsychological and interventional basis for BECTS.

## Data Availability Statement

The original contributions presented in the study are included in the article/supplementary material, further inquiries can be directed to the corresponding authors.

## Ethics Statement

This study was approved through the Medical Ethics Committee of the Affiliated Hospital of Zunyi Medical College, and written informed consent was obtained from all participants or their guardians after a complete description of the required measurements. Written informed consent was obtained from the individual(s), and minor(s)' legal guardian/next of kin, for the publication of any potentially identifiable images or data included in this article.

## Author Contributions

TZ and SL contributed to the conception, design, and radiological expertise, helped to select and assess cases, conducted the data analysis, and drafted and approved the final manuscript. LJ and XM contributed to the drafting and revision of the manuscript. JW and JZ offered data collection. HL and GZ contributed radiological expertise and offered a critical review of the manuscript for intellectual content. All the authors have read and approved the final manuscript.

## Funding

This study was supported by the National Natural Science Foundation of China (Grant Nos. 82160328, 82171919, and 81960312), Guizhou Provincial Natural Science Foundation (Project no. Qiankehejichu-ZK [2021] yiban 479), Natural Science Foundation of Zunyi (Project no. Zunshikehe HZ zi (2020) 143hao) and The First People's Hospital of Zunyi Yanjiuyushiyanfazhan R&D (Project no. yuankezi(2020) 9 hao).

## Conflict of Interest

The authors declare that the research was conducted in the absence of any commercial or financial relationships that could be construed as a potential conflict of interest.

## Publisher's Note

All claims expressed in this article are solely those of the authors and do not necessarily represent those of their affiliated organizations, or those of the publisher, the editors and the reviewers. Any product that may be evaluated in this article, or claim that may be made by its manufacturer, is not guaranteed or endorsed by the publisher.
